# MK-801 and cognitive functions: Investigating the behavioral effects of a non-competitive NMDA receptor antagonist

**DOI:** 10.1007/s00213-023-06454-z

**Published:** 2023-09-19

**Authors:** Anna Janus, Klaudia Lustyk, Karolina Pytka

**Affiliations:** Department of Pharmacodynamics, Faculty of Pharmacy, Jagiellonian University Medical College, Medyczna 9, 30-688 Krakow, Poland

**Keywords:** MK-801, Dizocilpine, Cognitive impairment, Behavioral research

## Abstract

**Rationale:**

MK-801 (dizocilpine) is a non-competitive NMDA receptor antagonist originally explored for anticonvulsant potential. Despite its original purpose, its amnestic properties led to the development of pivotal models of various cognitive impairments widely employed in research and greatly impacting scientific progress. MK-801 offers several advantages; however, it also presents drawbacks, including inducing dose-dependent hyperlocomotion or ambiguous effects on anxiety, which can impact the interpretation of behavioral research results.

**Objectives:**

The present review attempts to summarize and discuss the effects of MK-801 on different types of memory and cognitive functions in animal studies.

**Results:**

A plethora of behavioral research suggests that MK-801 can detrimentally impact cognitive functions. The specific effect of this compound is influenced by variables including developmental stage, gender, species, strain, and, crucially, the administered dose. Notably, when considering the undesirable effects of MK-801, doses up to 0.1 mg/kg were found not to induce stereotypy or hyperlocomotion.

**Conclusion:**

Dizocilpine continues to be of significant importance in preclinical research, facilitating the exploration of various procognitive therapeutic agents. However, given its potential undesirable effects, it is imperative to meticulously determine the appropriate dosages and conduct supplementary evaluations for any undesirable outcomes, which could complicate the interpretation of the findings.

## Introduction

Pharmacological induction of cognitive deficits in animals is an important model for human cognitive impairments (van der Staay et al. 2011). N-methyl-D-aspartate (NMDA) receptor dysfunction significantly contributes to cognitive deficits in various neurological and psychiatric disorders, including schizophrenia, depression, ischemic brain injury, and chronic neurodegenerative diseases (Zhou and Sheng 2013). Although considerable progress has been made in understanding these diseases’ mechanisms, there is still an emerging need to discover novel therapeutics and treatments. Using animal models is essential in this process, and in the context of NMDA receptors, MK-801-induced memory deficiencies are especially popular and useful for studying novel therapeutics. In addition to the memory disruptions, MK-801's ability to elicit effects like stereotypy and hyperlocomotion made it a valuable pharmacological model of schizophrenia, accurately mimicking positive and cognitive symptoms of this neuropsychiatric disorder (Hiyoshi et al. 2014; Latysheva & Rayevsky 2003; Martin et al. 1997; Smith et al. 2011; Svoboda et al. 2015). However, the MK-801-induced hyperlocomotion can pose challenges in research focused on specifically modeling memory impairments, as it can potentially influence the results (Liu et al. 2017a, b). Unfortunately, a significant number of published papers fail to address this issue, leading to the question of whether the observed memory impairment effects are independent of this side effect. Moreover, MK-801 has ambiguous effects on anxiety, which is another issue addressed herein. In this review, we critically assess the behavioral studies utilizing MK-801 as a model for cognitive impairments, considering the controversies despite its extensive use. Additionally, this paper attempts to summarize and discuss current knowledge about dizocilpine and experimental approaches taken by researchers in behavioral studies, with particular emphasis on its effects on different types of memory and cognitive functions.

## MK-801: effects on NMDA receptors and molecular properties

Dizocilpine maleate, also known as MK-801 ([( +)-5-methyl-lO,ll-dihydro-5H-dibenzo[a,d]cyclohepten-5,10-imine maleate]) (Javitt and Zukin 1989), is a non-competitive antagonist of synaptic NMDA receptor, widely used in behavioral studies. This compound has been initially tested as an anticonvulsant drug with potential anxiolytic effects (Sircar et al. 1987), as well as a neuroprotective agent in the cerebral ischemia model (Gill et al. 1987). Nowadays, dizocilpine is being extensively used in drug discovery studies to investigate the pharmacological effects of new drug candidates in rodent models of cognitive impairments induced by its acute or chronic administration (Ahlander et al. 1999; Gallant et al. 2017). Furthermore, MK-801 can also be exploited to examine the role of extrasynaptic NMDA receptors. When combined with low-frequency stimulation, dizocilpine selectively blocks only synaptic NMDA receptors (A. Z. Harris and Pettit 2007). MK-801 was also shown to induce neuronal degeneration (Kovacic and Somanathan 2010).

NMDA receptors, targeted by MK-801, are a critical component of excitatory transmission in the central nervous system. They belong to the family of ionotropic glutamate receptors and form heterotetramers composed of two GluN1 and two GluN2 subunits (Lorente et al. 2014). The architecture of NMDA receptors is domain-layered, involving an amino-terminal domain and ligand or agonist binding domain that reside in the synaptic space, as well as a transmembrane domain that spans the membrane (Karakas and Furukawa 2014; C.-H. Lee et al. 2014). Moreover, NMDA receptors are so-called ‘coincidence detectors’ due to their very characteristic property of being activated only when depolarization occurs and two glutamate molecules are bound. This phenomenon results from the fact that NMDA receptors have a magnesium block in the structure of their ion channel, which prevents their activation, even though two molecules of an agonist are bound (Mayer et al. 1984).

NMDA receptors play an important role in numerous forms of synaptic plasticity and various aspects of development and synaptic transmission (Hardingham and Bading 2010). Furthermore, these receptors are widely spread within the brain structures, although the location of different subunits varies, e.g., the NMDAR2A and NMDAR2B mRNAs are prominently expressed in the forebrain (Nakanishi 1992), whereas the NMDAR2C and NMDAR2D mRNAs are predominantly expressed in the cerebellum and the diencephalic/lower brain stem regions, respectively (Nakanishi 1992). Activation of NMDA receptors can trigger both long- and short-term plasticity, promote cell survival, and initiate cell death (A. Z. Harris and Pettit 2007). Moreover, the involvement of NMDA receptors in the long-term potentiation (LTP) processes in the hippocampus is crucial, as evidenced by the behavioral effects caused by its antagonist MK-801, which is directly associated with mechanisms underlying memory formation (Cole et al. 1989). Among the group of NMDA receptors phencyclidine-like antagonists, MK-801 and ketamine are devoid of subunit selectivity (Dix et al. 2010) and their mechanism of action is dependent both on the dosage and the applied voltage (Lodge & Johnson, 1990). MK-801, as a noncompetitive NMDA receptor-channel blocker binds to sites within the NMDA receptor channel pore, like ketamine and memantine (Ellison 1995). Crystallography research indicates that MK-801, by targeting the transmembrane domain of the NMDA receptor, binds within the ion channel vestibule and promotes its closure (Song et al. 2018). This study suggests that by entering its binding site through an open, activated, intact receptor, MK-801 acts via the aqueous path, physically blocking ion permeation (Song et al. 2018). Interestingly, the thriving popularity of MK-801 in behavioral studies is tied to the fact that it effectively mimics various symptoms of neuropsychiatric disorders in a dosage-dependent manner (Mabunga et al. 2019). Furthermore, ion channel blockade caused by dizocilpine is essentially irreversible throughout most experiments (Hardingham and Bading 2010). Additionally, a vast advantage of MK-801 is its high affinity to NMDA receptor (circa 3 nM), as well as selectivity (Reynolds et al. 1987). Song et al. discovered that (Song et al. 2018) this high affinity and long dwell time of dizocilpine are the cause of side effects that exclude its clinical application as a therapeutic for excitotoxicity-related disorders.

A large body of research investigating the cellular targets of MK-801 has focused primarily on schizophrenia models, with a particular interest in NMDA receptors located on cortical parvalbumin immunoreactive (PV +) interneurons. In the paper of Lisman et al. (2008), it was suggested, that NMDA antagonists reduce the excitation of fast spiking interneurons leading to the disinhibition of pyramidal cells. As showed in the study of Homayoun and Moghaddam (2007) NMDA receptor inhibition by MK-801, results in indirect excitation of pyramidal neurons, through disinhibition of GABAergic neurons. The study of Xi et al. (2009) suggested, that PV + interneurons are highly sensitive to MK-801 stimulation, and even low dose of the compound caused significant decreases of NMDA receptor subunits on these neurons, whereas the same doses induce the increase in pyramidal neurons in prefrontal cortex. Moreover, it was recently showed that subchronic administration of MK-801 results in the reduction of PV + and the calbindin-containing GABAergic interneurons in the hippocampus and medial prefrontal cortex (Tsai et al. 2022). Nonetheless, further investigations are required to unravel the specific cellular targets that contribute to MK-801's amnesic effects.

The behavioral effects of MK-801 differ depending on the animal strain, sex, developmental stage and the timing of administration (Mabunga et al. 2019). Moreover, dose-dependent hyperlocomotion induction is, as we have already mentioned, one of the most problematic aspects during the experiments (Martin et al. 1997). This effect also appears after systemic injection of MK-801 and, at higher doses, can lead to stereotypic behaviors, such as head waving and uncoordinated ataxic gait (Clineschmidt et al. 1982; S I Deutsch et al. 1997). MK-801 induces cognitive impairments without causing sensory, locomotor, or toxicological side effects in a small dose range (van der Staay et al. 2011), which we have addressed in the following chapters of our review.

Intra-hippocampal and intra-prefrontal cortex administration of MK-801 shows differential effects on the central nervous system (Foster et al. 1988; López-Gil et al. 2012; Nowak et al. 2012; Schurr et al. 1995). MK-801’s treatment of hippocampal slices that were exposed to hypoxia, showed that this drug effectively protected slices against hypoxic neuronal damage (Schurr et al. 1995). However, these effects of MK-801 also raised some controversies and other studies indicated that the protective effects may result from post-ischemic hypothermia (Buchan and Pulsinelli 1990; Hara et al. 1992). In the publication of Moyanova et al. (2007) it was pointed out, that MK-801 may be useful as a reference drug for safer neuroprotective compounds in the model of focal ischemia. Interestingly, recent findings showed that intrahippocampal administration of MK-801 facilitated memory acquisition, whereas systematic injection produced contrasting effects in mice (Hsiung et al. 2020). MK-801 facilitated memory consolidation and retrieval in both intrahippocampal and systemic administration routes, and it was suggested that the enhancement of memory consolidation could be caused by an increase in mTOR phosphorylation (Hsiung et al. 2020). On the contrary, another study demonstrated that intra-ventral-hippocampal administration caused spatial memory impairments in rats (Xu et al. 2005). Moreover, it was recently indicated that infusion of MK-801 into the medial prefrontal cortex increased the levels of serotonin, noradrenaline, and dopamine while decreasing the level of GABA (Okada et al. 2019). Conversely, earlier studies showed that intra-prefrontal cortex infusion of this compound does not affect serotonin levels. Similarly, behavioral studies present contraindicatory findings. For instance, an intra-prefrontal cortex infusion of MK-801 has been shown to induces stereotypy (Nowak et al. 2012). Conversely, other studies have suggested that hyperlocomotion and stereotypy can be caused only by systemic administration, demonstrating no observable changes in the behavior of animals treated with MK-801 intra-hippocampally (López-Gil et al. 2012).

## Effects of MK-801 on memory in animal studies

MK-801 has been studied in the context of memory impairments since the publication of Benvenga and Spaudling's research (Benvenga and Spaulding 1988), which showed that dizocilpine induces memory impairments. This study indicated that MK-801 at doses 0.1 and 0.15 mg/kg produced an amnestic effect and increased locomotor activity in male mice. Therefore, it laid the basis for further research on memory impairments caused by MK-801.

### MK-801 and recognition memory

Recognition memory can be defined as the ability to detect the prior occurrence of a given stimulus, which is an inherent aspect of declarative memory. This type of memory relies on several neurochemical pathways involving glutamatergic pathways (Cho et al. 2000). Activating these pathways by novel objects can lead to synaptic plasticity processes that reduce neuronal responses during future encounters, thus enabling recognition (Cho et al. 2000). It is believed that major structures engaged in aspects of recognition memory are the perirhinal cortex, hippocampus, temporal association cortex, and medial prefrontal cortex, according to the Warburton et al. (2013) review. Behavioral paradigms that allow the examination of spontaneous recognition memory are well established, and the most popular tasks include object recognition, object location, and temporal object location. All the abovementioned tests explore animals’ natural preference for novelty, which makes them exceptionally useful in studies on the physiology and pathology of memory. Recognition memory tests allow the investigation of memory impairments, which often occur in neurodegenerative, psychiatric, and autism spectrum disorders (Cruz-Sanchez et al. 2020), as well as test compounds with procognitive potential.

Several studies using MK-801 explored its effects on the retention of recognition memory while treating animals before or after training. For instance, De Lima et al. (De Lima et al. 2005) showed that both pre- and post-training intraperitoneal (*ip)* administration of 0.01 and 0.1, but not 0.001 mg/kg of MK-801 impaired formation of memory for short-term and long-term retention in the object recognition task in adult female Wistar rats. Additionally, the compound had its most significant effect when it was administered before the encoding phase. Another study showed that *ip* administration of MK-801 at doses 0.1 or 0.2 mg/kg while pre-training decreased novel object exploration time, and thus encoding of recognition memory in NMRI adult male mice (M. Nilsson et al. 2007). Surprisingly, post-training and pre-recognition session systemic treatment significantly increased interest in a novel object during the recognition session. However, as suggested by the authors, the increased novel object exploration time was presumably attributable to the psychotogenic properties of MK-801 rather than its potential for memory enhancement. An additional hypothesis posits that MK-801 induced an anxiolytic-like effect, thereby promoting neophilic behaviors in animals (M. Nilsson et al. 2007). Furthermore, authors pinpointed that observed memory consolidation facilitation may be somehow associated with the NMRI strain (M. Nilsson et al. 2007). Conversely, the research on female C57BL/6 J mice argued against the hypothesis and examined whether MK-801, instead of impairing memory encoding, may act state-dependently and whether the change in a drug-state causes impairments (M. Chan et al. 2019). In the first part of this study, animals received either MK-801 (0.01 mg/kg, *ip*) or vehicle before the exposure phase on day 1, and then 24 h later, they were administered with either the same or different substance prior to the test phase. The results showed that MK-801 impaired memory in a state-dependent way, while a compound did not actually affect memory encoding and retrieval. In the second experiment, animals underwent identical procedure; however, they were administered with a higher MK-801 dose (0.1 mg/kg), and interestingly, mice that received MK-801 prior to both encoding and test phase did not show recognition memory deficits. It was also reported that MK-801 induced locomotor hyperactivity in a higher dose. Moreover, according to the authors, the period between the exposure and test phases (1 day), as well as the lack of memory deficits, may suggest that MK-801 impaired short-term but not long-term recognition memory (M. Chan et al. 2019). However, comparing these contrasting results is challenging due to the fact that this study was conducted on female mice, and the potential influence of the estrus cycle phase on the MK-801-induced effects cannot be overlooked.

The influence of MK-801 on recognition memory can also be investigated in different species. Recent advances in various neuroscientific methods allowed for the development of cognitive impairments models in zebrafish. Gaspary et al. (2018) tested recognition memory deficits caused by MK-801 (5 µM or 10 µM) in zebrafish. The scientists discovered impairments in recognition memory using the object recognition and object location paradigms; however, it is worth mentioning that the stress-inducing novel environment could have influenced this result (Gaspary et al. 2018). Another attempt to develop MK-801-induced recognition memory impairments in non-rodent models was conducted on adult marmosets (Oliveira et al. 2021). This study showed that acute, systemic administration of MK-801 (0.015 mg/kg) impaired recognition memory in the object recognition test, and as the authors noted, this result was unlikely associated with changes in locomotor activity or overall exploration. Additionally, a study on domestic rabbits showed that MK-801 (37 µg/kg) negatively influenced recognition memory assessed in the object recognition test (Hoffman & Basurto 2013). However, findings suggest that it only impacted short-term habituation and sensitization rather than long-term memory, as this effect was not observed with delays longer than 5 min.

To sum up, abovementioned studies suggest that MK-801 may affect recognition memory encoding and consolidation in a different manner, however these effects may be influenced by sex and strain of the animals. In male mice, MK-801 impaired recognition memory encoding, however, its impact on memory consolidation appeared to be absent, possibly due to the presence of side effects. The studies conducted on female specimens showed conflicting results, i.e., while dizocilpine impaired both memory consolidation and encoding in Wistar rats, it had no effect on either of these processes in C57BL/6 J mice. The literature investigating the effects of acute MK-801 administration on memory encoding and consolidation is relatively limited. Therefore, it is important to address this issue in future studies in order to draw clear conclusions on this matter.

### MK-801 and spatial memory

Spatial memory refers to the ability to learn and remember spatial locations and associate them with other stimuli (Bannerman et al. 2014). The processes underlying this type of memory should involve learning, consolidation, storage, and later retrieval of a spatial memory trace (Zorzo et al. 2022). According to the theory of O’Keefe and Nadel (John O’Keefe and Nadel 1979), spatial memory can be divided into two types: egocentric, which relates to the ability to remember the object's location in relation to one’s own position, and allocentric, which requires encoding of the spatial relationship among objects in the environment. Furthermore, another spatial memory division distinguishes spatial reference and working memory. It is believed that an allocentric spatial memory is being maintained in the dorsal hippocampus (CA1 subfield), although recent studies indicate that it is associated with distributed neural circuit that goes beyond the hippocampus (Rinaldi et al. 2020). Despite this anatomical ambiguity, it was discovered that the hippocampus consists of very characteristic, glutamatergic place cells, which increase their firing rate when an organism is located in a certain place in the environment (J O’Keefe & Conway 1978). This electrophysiological pattern appears specific to the location (J O’Keefe & Conway 1978). Moreover, as described in Bannerman et al. (2014) recent studies have shown that there are also several different cell types encoding different associated spatial features, including grid cells (Fyhn et al. 2004), boundary vector cells (Savelli et al. 2008), and head direction cells (Taube et al. 1990). Spatial memory is being widely tested in behavioral studies using different paradigms, including a very popular Morris water maze (Morris et al. 1990), T-maze (Lalonde 2002), or radial arm maze (Schmitt et al. 2003).

A significant body of evidence indicated that MK-801 impairs spatial memory in the Morris water maze (Åhlander et al. 1999; Enomoto et al. 2008; Mutlu et al. 2011; Ning et al. 2017; O’Donnell et al. 2003). However, different studies use different routes of administration of this compound. According to a study by Svalbe et al., despite the lipophilic character of dizocilpine, the vast majority of studies use *ip* route of MK-801 administration rather than subcutaneous (*sc)* (Svalbe et al. 2019). However, mentioned research suggested that *sc* injections were more effective than *ip* in impairing spatial learning and memory, which was examined using male Wistar rats in the Morris water maze. The *sc* administration of MK-801 (0.01, 0.05, 0.1 mg/kg) caused its higher blood and brain tissue concentrations compared to other routes. Additionally, it impaired memory at 0.05 and 0.1 mg/kg doses, but this effect was not observed at the dose of 0.01 mg/kg. It is worth mentioning that the dose of 0.1 mg/kg caused hyperlocomotion regardless of the administration route (Svalbe et al. 2019).

A single, acute administration of a high MK-801 dose (5 mg/kg, *ip*) in male Wistar rats performing radial maze resulted in deficits of reference but not working spatial memory, and LTP impairment appeared 7 days and 4 weeks after the injection (Manahan-Vaughan et al. 2008). Moreover, animals exposed to such a high dose of MK-801 also exhibited stereotypy, ataxia, and hyperlocomotion, although these effects typically disappear within 24 h. The observed symptoms make the systemic administration of high doses of MK-801 a model for a single psychotic episode in schizophrenia (Stephen I Deutsch et al. 2002). MK-801 has also been extensively studied for its effects on neural development using chronic administration of lower doses and it is described in the section dedicated to developmental correlates of MK-801.

Referring to research on other species, a study conducted on goldfish by (Gómez et al. 2006) indicated that intracranial administration of MK-801 into the telencephalon, which is thought to reflect mammal hippocampal spatial memory system, impaired spatial learning and memory in a dose-dependent manner (Gómez et al. 2006). However, in this study, neither the sex, nor developmental stage of specimens was mentioned, even though these factors are significant and may influence the results. Another study conducted on adult male zebrafish, showed that MK-801 (20 µM) dissolved in tank water caused spatial memory deficits, which were assessed using the Y-maze paradigm (Cognato et al. 2012) Finally, it was discovered that dizocilpine at the dose of 0.02, but not 0.005 mg/kg, administered intramuscularly in adult male rhesus monkeys caused impairments in spatial memory retrieval (Wang et al. 2007).

In brief, acute, systemic injection of MK-801 was showed to successfully impair spatial memory assessed in Morris water maze in Wistar rats. Importantly, as highlighted in Svalbe et al. (2019) *sc* route of administration may induce this effect more effectively. Interestingly, a high dose of MK-801 (5 mg/kg) negatively influenced long-term memory in Wistar rats tested in the radial arm maze, while short-term memory remained unaffected.

MK-801 was also indicated to impair both spatial and recognition memory in non-rodent species, such as fish or primates, suggesting that the memory-impairing effects of NMDA antagonism may be conserved across different species. The effects of MK-801 on recognition and spatial memory are summarized in Tables 1 and 2, respectively.

**Table 1:** Effects of MK-801 on recognition memory in various behavioral tests in animals

Object recognition test								
Dose (mg/kg)	Administration route	Acute adm	Chronic adm	Species	Sex	Developmental stage on the day of experiment	Memory deficits	Reference
0.1	*sc*	-	GD 7–19	Long Evans rats	M	juvenile	-	(Gallant et al. 2017)
						adult	+	
0.25	*sc*	-	P5-14	Sprague–Dawley rats	M	adolescent	+	(J. T. Li et al. 2015)
						adult	+	
0.1	-	-	P7-P21	Sprague–Dawley rats	M	adolescent	-	(Liu et al. 2017a, b)
0.3							-	
0.5							+	
0.2	*ip*	-	P7-10	Sprague–Dawley rats	M	adult	-	(Lim et al. 2012)
	*ip*	-	P7-10 + IR				+	
0.05	*ip*	-	P28-41 + 24 h	Sprague–Dawley rats	M	adult	-	(Ji Tao Li et al. 2011)
0.1							-	
0.2							-	
0.05	*ip*	-	P28-41 + 14 days				-	
0.1	*ip*	-	P7-14	C57BL/6 mice	M	adult	+	(Plataki et al. 2021)
					F	adult	+	
	*ip*	-	P11-16		M	adult	±	
					F	adult	±	
1	*sc*	-	P7	Swiss albino mice	M	adolescent	+	(Kawade et al. 2020)
	*sc*	-	P14				+	
	*sc*	-	P21				+	
1	*sc*	-	P7	Swiss albino mice	M	adult	+	(Kawade et al. 2020)
	*sc*	-	P14				+	
	*sc*	-	P21				+	
0.01	*ip*	+	-	Wistar rats	F	adult	+	(De Lima et al. 2005)
0.1							+	
0.1	*ip*	+	-	NMRI mice	M	adult	+	(M. Nilsson et al. 2007)
0.2							+	
0.01	*ip*	+	-	C57BL/6 J mice	F	adult	-	(Chan et al. 2019)
0.1							-	
5 µM	dissolved in tank water	+	-	zebrafish	M	adult	+	(Gaspary et al. 2018)
10 µM							+	
0.015	*sc*	+	-	marmoset	M	adult	+	(Oliveira et al. 2021)
					F		+	
0.037	*sc*	+	-	domestic rabbit	M	adult	+	(Hoffman and Basurto 2013)

adm. – administration*, sc* – subcutaneously, *ip* – intraperitoneally, GD7-19 – gestational day 17–19, P5-14 – postnatal day 5–15, P7-14 – postnatal day 7–14, P11-16 – postnatal day 11–16, P7-21 – postnatal day 7–21, P7-10 – postnatal day 7–10, P28-41—postnatal day 28–41, P7 – postnatal day 7, P14 – postnatal day 14, P21 – postnatal day 21, IR – isolation rearing, M-male, F-female, -no, + yes, ± yes and no

**Table 2:** Effects of MK-801 on spatial memory in various behavioral tests in animals

Dose (mg/kg)	Administration route	Acute adm	Chronic adm	Species	Sex	Developmental stage on the day of experiment	Memory deficits	Reference
1. Radial arm maze								
5	*ip*	+	-	Wistar rats	M	adult	+	(Manahan-Vaughan et al. 2008)
0.4	*sc*	-	P7-13	Wistar rats	M	adult	+	(Furuie et al. 2019)
	*sc*	-	P14-20				-	
	*sc*	-	P7-20				+	
2. Morris Water Maze								
0.2	*ip*	+	-	Balb-c mice	M	adult	+	(Mutlu et al. 2011)
0.1	*ip*	+	-	C57BL/6 mice	M	adult	+	(Ning et al. 2017)
0.01	*ip*	+	-	Wistar rats	M	adult	-	(Svalbe et al. 2019)
0.05							+	
0.1							+	
0.01	*sc*	+	-				-	
0.05							+	
0.1							+	
0.4	*sc*	-	P7-13	Wistar rats	M	adult	±	(Furuie et al. 2019)
	*sc*	-	P14- 20				-	
	*sc*	-	P7-20				+	
0.25	*ip*	-	28 days	Wistar rats	M	adult	-	(J.-T. Li et al. 2013)
0.5	*sc*	-	P30-43	Wistar rats	M	adult	-	(Uttl et al. 2018)
	*sc*	-	P60-73				-	
0.5	*sc*	-	P30-43	Long-Evans rats	M	adult	-	(Uttl et al. 2018)
	*sc*	-	P60-73				+	
3. Elevated plus maze								
1 µg	*ic*	+	-	goldfish	n/a	n/a	-	(Gómez et al. 2006)
2.5 µg							+	
4 µg							+	
4. Y-maze								
20 µM	dissolved in tank water	+	-	zebrafish	M	adult	+	(Cognato et al. 2012)
5. Spatial memory task for monkeys								
0.005	*im*	+	-	rhesus monkey	M	adult	-	(Wang et al. 2007)
0.02							+	

adm. – administration*, sc* – subcutaneously, *ip* – intraperitoneally, *ic* – intracerebrally, *im* – intramuscularly, P7-13 – postnatal day 7–13, P14-20 – postnatal day 14–20, P7-20 – postnatal day 7–20, P30-43 – postnatal day 30–43, P60-73 – postnatal day 60–73, M-male, F-female,- no, + yes, ± yes and no, n/a- no applicable information

## Effects of MK-801 on emotional conditioning in animal studies

Emotional memory in humans can be defined as the learning and storage of information about the emotional significance of experiences (LeDoux 1993), and it is believed that this processing has an adaptive character. Equivalently, this type of memory is behaviorally tested in rodents using fear conditioning paradigms.

Pavlovian fear conditioning is a widely known procedure used in behavioral studies in the context of memory since it was discovered that such emotions could be rapidly memorized (Watson & Rayner 1920). The pairing of unconditional stimulus, which can be either aversive or rewarding, with a neutral conditional visual or auditory stimulus results in the association of conditional stimulus with a rewarding or aversive event and a behavioral response to this stimulus even in the absence of an unconditional stimulus. Furthermore, the neural basis of this process is believed to be associated with the activity of the dopaminergic system. Schultz et al. (1997) in his groundbreaking study showed that ventral tegmental area dopaminergic neurons react to rewarding conditional stimulus with a phasic increase in the activity and with phasic inhibition to aversive conditional stimulus. Behavioral experiments on fear conditioning in rodents usually include a pairing of conditional stimulus (e.g. tone sound) with a foot shock, and this procedure results in conditioned freezing in rats or freezing/tachycardia in mice (Zambetti et al. 2022). Importantly, this phenomenon results in learning, which is some kind of adaptation for animals to avoid a specific aversive event in the future or easier obtain a reward. In the case of fear conditioning, this associative learning depends on the hippocampus and amygdala (Phillips et al. 2003).

Considering fear memory, aside from the conditioning paradigms, the passive avoidance conditioning task is a prevalent paradigm widely used in behavioral studies. In this task, the animal associates a particular environment (a specific part of a chamber) with an aversive event (e.g., foot shock) and uses this information to avoid this ‘aversive’ compartment in the subsequent sessions. The smaller the delay in the animal moving to this compartment, the more disrupted its emotional memory.

### MK-801 and passive avoidance conditioning

A considerable amount of research shows that MK-801 causes impairments in the passive avoidance conditioning task in rodents while administered prior to the testing procedure (Mondadori and Weiskrantz 1993; Venable and Kelly 1990). Accordingly, adult Wistar rats administered with MK-801 (0.08, 0.1 mg/kg, *sc*) before training showed impairments in the retention test, which suggests that MK-801 disrupted the consolidation process (van der Staay et al. 2011). However, a lower dose of MK-801 (0.05 mg/kg) did not affect the tested animals (van der Staay et al. 2011). A different study investigating the effects of MK-801 at varying doses using the same paradigm, showed that dizocilpine impaired memory consolidation in Wistar rats most effectively at the dose of 1.125 mg/kg (Monteiro Moreira et al. 2005). Researchers suggest that the effect of MK-801 on consolidation and reconsolidation in the passive avoidance conditioning paradigm may be state-dependent (Ceretta et al. 2008; Flint et al. 2013; Harrod et al. 2001). Adolescent Sprague–Dawley rats, treated/ with MK-801 (0.1 mg/kg, *ip*) immediately after training, had impaired memory consolidation and a shorter retrieval trial latency than the control group (Flint et al. 2013). In this study, the researchers also tested the effect of dizocilpine on reconsolidation by conducting short reactivation procedure. A group of animals was placed in the non-aversive side of apparatus, and immediately after that, they received MK-801 or saline, while another group received MK-801 without a reactivation procedure. Interestingly, the results showed that only the group that received MK-801 after the reactivation procedure had impaired retention test performance. In the subsequent experiment, animals were divided into four groups and underwent training. Then, immediately after the reactivation phase, two groups received MK-801 and the other two – saline. In the last phase of the test, all groups were treated with MK-801 or saline 20 min prior to the retention phase. The results showed that groups saline/saline and MK-801/MK-801 performed better in the retention test than groups saline/MK-801, MK-801/saline. This finding indicates that MK-801 is state-dependent in its mechanism of action (Flint et al. 2013). The described results are in line with another study (Ceretta et al. 2008), that investigated whether a relatively low dose of MK-801 (0.03 mg/kg, *ip*), and arcaine (competitive antagonist of the polyamine binding site at the NMDA receptor) would impair performance in a similar task in a dose-dependent manner. This study used the one-trial step-down inhibitory avoidance paradigm to assess the compound's impact on memory. The task consisted of a training phase, in which animals were placed on a platform situated within a training apparatus. If an animal stepped down from the platform, it would immediately receive a footshock. In the testing phase, that was carried out 24 h after training, the platform step-down latency was measured to assess memory retention. To investigate the effect of MK-801, animals were divided into seven groups, each receiving two injections – one immediately after training and the second 30 min before testing in the following combinations: PBS/PBS, arcaine/PBS, arcaine/MK-801, arcaine/arcaine, MK-801/PBS, MK-801/MK-801 and MK-801/arcaine. The results indicated that both compounds acted state-dependently (Ceretta et al. 2008).

### MK-801 and classic conditioning

Many studies reported that MK-801 impairs memory in cued (hippocampus-independent) or contextual (hippocampus-dependent) fear conditioning (Bardgett et al. 2003; Gould et al. 2002; Langton et al. 2007). For instance, bilateral infusion of MK-801 (6.25 µg/side) into the ventral hippocampus of Wistar rats before the fear conditioning impaired contextual learning (Zhang et al. 2001). Furthermore, the study of Seemiller and Gould (2021) examined whether sex, age, as well as genetic profile, influence drug-induced learning deficits using these types of conditioning. This study was conducted on two strains of mice, C57BL/6 J and DBA/2 J, and MK-801 affected cued and contextual learning in classical fear conditioning. MK-801 (0.05 or 0.01 mg/kg, *ip*) was administered before the training, and the memory retention was examined one week later. It was observed that adolescent C57BL/6 J mice were more sensitive to MK-801 in contextual learning, and generally, MK-801 had a greater impact on contextual rather than cued learning (Seemiller and Gould 2021). Moreover, another study reported that MK-801 (0.3, 0.1 mg/kg, *sc*) administered prior to the training in F344 male rats impaired contextual but not cued conditioning (Gould et al. 2002). Interestingly, post-training treatment neither affected contextual nor cued conditioning, and it was even suggested that a dose of 0.3 mg/kg enhanced cued conditioning (Gould et al. 2002). MK-801 was tested for its effects on fear conditioning and associative learning also in zebrafish using classical fear conditioning paradigm, where the electric shock served as an aversive stimulus (Kenney et al. 2017). In this investigation, MK-801 (20 µM) was administered following an acquisition of fear conditioning procedure and was shown to prevent the conditioning (Kenney et al. 2017).

The abovementioned studies indicate that MK-801 can successfully impair memory consolidation when administered prior to the passive avoidance paradigm. However, ambiguity in the results depending on the state was reported, indicating potential state-dependent effects of MK-801. Moreover, MK-801-induced NMDA receptors blockade effectively impairs contextual, but not cued fear conditioning, which suggests that mechanisms underlying these two types of learning differ. The studies that are discussed in this section are presented in Table 3.

**Table 3:** Effects of MK-801 on emotional/aversive memory in various behavioral tests in animals

Dose (mg/kg)	Administration route	Species	Sex	Developmental stage on the day of experiment	Memory deficits	Reference
1. Passive avoidance conditioning						
0.05	*sc*	Wistar rats	M	adult	-	(van der Staay et al. 2011)
0.08					+	
0.1					+	
2. Contextual fear conditioning						
6.25 µg/side	*ic*	Wistar rats	M	adult	+	(Zhang et al. 2001)
0.05	*ip*	C57BL/6 J mice	F	adolescent	+	(Seemiller and Gould 2021)
			M		+	
			F	adult	-	
			M		-	
		DBA/2 J mice	F	adolescent	-	
			M		-	
			F	adult	-	
			M		-	
0.1	*ip*	C57BL/6 J mice	F	adolescent	+	
			M		+	
			F	adult	+	
			M		+	
		DBA/2 J mice	F	adolescent	-	
			M		-	
			F	adult	-	
			M		-	
20 µM	dissolved in tank water	AB zebrafish		adult	+	(Kenney et al. 2017)
		Tu zebrafish			+	
		TL zebrafish			+	
0.1	*sc*	F344 rats	M	adult	+	(Gould et al. 2002)
0.3					+	
3. Cued fear conditioning						
0.1	*sc*	F344 rats	M	adult	-	(Gould et al. 2002)
0.3					-	

*sc* – subcutaneously, *ic—*intracerebrally, *ip* – intraperitoneally, M-male, F-female, -no, + yes

## Effects of MK-801 on executive functions in animal studies

Human executive functions can be generally described as a complex of different high–level cognitive processes that enable individuals to regulate their thoughts and actions during the goal–directed behavior through their influence on lower–level processes (Friedman & Miyake 2017). These functions include planning, working memory processes, switching between tasks or response inhibition, and their general role is to effectively implement goal–directed actions as well as to control attentional resources (Yogev-Seligmann et al. 2008). A number of diseases and psychiatric disorders are associated with deficits in executive functions, such as impairments of working memory or behavioral flexibility reported in Alzheimer’s disease (Baudic et al. 2006), schizophrenia (Wobrock et al. 2009), depression (Rogers et al. 2004), Parkinson’s disease (Millan et al. 2012), autism (Ozonoff et al. 1991) or substance abuse (Koob and Volkow 2016). The prefrontal cortex and its circuity are believed to play a central role in cognitive executive functions (Joaquín M Fuster 2019). However, evidence indicates that the prefrontal cortex exhibits a disproportionate growth compared to other cortical regions throughout evolution. In humans, it constitutes 29% of the total cortex, while in chimpanzees it accounts for 17%, and in cats for only 3,5% of total cortical volume (Joaquín M Fuster 2002). This disproportion of the human prefrontal cortex over other species raised questions about whether its function can be measured in other species. This aspect becomes more controversial in rodents, as the ongoing debate questions whether rodents actually possess a prefrontal cortex (Laubach et al. 2018). Additionally, their less varied and adaptable behavioral repertoire adds to the complexity of this issue (Chudasama 2011). Despite inconsistencies, animal models of executive functions remain significant (Chudasama 2011). Importantly, rodent lesion studies indicate that their frontal cortex is indeed involved in executive functions (Birrell and Brown 2000; Granon et al. 1998). Behavioral rodent studies on executive functions involve tests and paradigms associated with working memory, behavioral flexibility, impulse control, and attention. A fundamental challenge in testing executive functions in animal models is the development and/or improvement of cross–species paradigms, along with tests that target similar behavioral dimensions or neurobiological measures across a range of species. This challenge was confronted in the study of Cleal et al. (2021). In the following section, we describe the effects of MK-801 on animals working memory, cognitive flexibility and attentional processing.

### MK-801 and working memory

The ability to implement a specific mechanism of action in a purposeful behavior requires both “holding” information about certain context, as well as using previously stored information associated with the specific situation. According to the review of Sheynikhovich et al. (2023) working memory depends on two distinct mechanisms: online control including processes like decision-making and active maintenance of goals or rules, which lasts over a duration of seconds to minutes, and long–term storage of contextual representations. The prefrontal cortex is believed to play a pivotal role in this type of memory and is thought to be a critical node in the network associated with central–executive subsystems (Castner et al. 2004). Two groundbreaking studies, which were a cornerstone in understanding cellular mechanisms underlying human and non-human primate working memory, were conducted by Goldman-Rakic (1995) and J M Fuster and Alexander (1971). They showed that the dorsolateral prefrontal cortex of primates locates cells that exhibit increased spiking when an animal is presented with a stimulus in a specific location within the visual field. Furthermore, even after the stimulus is gone, these neurons continue to spike at an elevated level. This discovery led to the anatomical debate about the structure responsible for working memory in rodents, since they do not have a prefrontal cortex per se, however some of the frontal cortical regions of the rodents cortex are thought to be equivalent to the primate prefrontal cortex (Laubach et al. 2018). Given that the medial prefrontal cortex in rodents is involved in numerous common function with the dorsolateral prefrontal cortex in primates, it is believed to be analogous (Dalley et al. 2004). Apart from the anatomical ambiguity, the working memory can be assessed in both rodents and non–human primates in tasks that involve spatial information such as spatial delayed response and spatial search tests (Chudasama 2011). It is worth highlighting that in rodents, working memory is mainly studied in the aspect of short–term memory.

The acute administration of MK-801 (0.12 and 0.15 mg/kg, *sc*) in male Long-Evans rats impaired working memory assessed in the allothethic place avoidance paradigm (Zemanova et al. 2013). However, in the group of animals that had undergone pretraining, this effect was absent, and it is worth mentioning that both groups exhibited hyperlocomotion. These results were consistent with another investigation (Novitskaya et al. 2010). A more recent study showed that a low dose of MK-801 (0.06 mg/kg, *sc*) also impaired working memory, but did not affect attentional processing in an automated touchscreen chamber in adult male Wistar rats (J. Lee et al. 2020). The study of MacQueen et al. (2011) investigated the effects of different doses of MK-801 (0.03, 0.1, 0.17, and 0.3 mg/kg, *ip*) in male Holtzman Sprague–Dawley albino rats on the performance in the odor span task. The results showed that MK-801 impaired test performance in a dose–dependent manner: doses 0.1 and 0.17 mg/kg affected negatively odor span task, but did not impair the simple discrimination task. Additionally, the deficits induced by the dose of 0.17 mg/kg were unrelated to memory load, whereas effects of 0.1 mg/kg dose appeared to be dependent on the number of stimuli that needed to be remembered (MacQueen et al. 2011). Similarly, more recent studies demonstrated that MK-801 (0.1 mg/kg) impaired odor span task, but not simple discrimination task, and that impairment was dependent on the memory load (Galizio et al. 2013, 2019).

### MK-801 and cognitive flexibility

The ability to behaviorally adapt is one of the most crucial survival skills for both animals and humans, especially in the face of the constantly changing external environment and it can be defined as cognitive flexibility. This executive function demands accompanying components, such as the inhibition of preceding responses, acquisition of new strategies, or attention to contextual changes (Prado et al. 2017). Processes that underlie cognitive flexibility depend on the effective engagement of different brain processes to select important cues from the environment properly, focus attention on it, determine the best strategy, and inhibit responses to the strategy that is not appropriate (S. R. O. Nilsson et al. 2015). The most popular paradigm to evaluate cognitive flexibility in humans is the Wisconsin card sorting test (Milner 1963), in which the participant must establish the classification principle through trial and error, which requires planning, guidance by personal representation, mental flexibility, and working memory. However, currently recommended tests include CANTAB intradimensional/extradimensional set-shifting (touchscreen technology) and switching Stroop tasks (Barch et al. 2009). According to the review of Guo et al. (2017), it is believed that at least part of the processes associated with cognitive flexibility are dependent on the activity of the frontal lobe, as well as cortical and subcortical sensory circuits. In rodents, cognitive flexibility is often tested in set–shifting task (Birrell and Brown 2000), in which animals learn to associate reward in a filled bowl according to its texture, the texture of the filling material, or its odor. This task requires reversals, as well as intradimensional and extradimensional shifts. The set-shifting paradigm is considered as ethologically appropriate, as it is analogous to the extended Wisconsin card sorting test but relies on olfaction or somatosensation and digging instead of vision and depends on the activity of the medial prefrontal cortex (Birrell and Brown 2000). The development of touchscreen technology, involving paradigms such as pairwise visual discrimination and reversal learning, allows for assessing behavioral flexibility in rodents and creates more complex and challenging tasks with improved reproducibility (Savolainen et al. 2021). In this method, the animal responds to the presented visual stimuli through a screen nose poke (Bussey et al. 2008).

The review of Uddin (2021) points out that there is another term that is often used interchangeably with the term ‘cognitive flexibility’ in the neuroscience literature, and it is ‘behavioral flexibility’ that can be defined as the ability to adaptably change the behavior in response to changing environmental contingencies. Studies on behavioral flexibility deficits, which are associated with various neuropsychiatric diseases, indicated that NMDA receptors are involved in these processes. In the following sub-sections, we described the findings on the effects of MK-801 on behavioral flexibility in rodents.

The influence of acute administration of MK-801 (0.1 mg/kg, *ip*) in adult, male Sprague–Dawley rats, was examined in the pairwise discrimination and reversal learning task, and the results suggested that it robustly impaired performance in this task (Mark R Stefani & Moghaddam 2010). Moreover, this effect was attenuated by the positive allosteric mGlu_5_ receptor modulator. Furthermore, Svoboda et al. (2015) used the rotating arena-based apparatus, that required avoiding an unmarked sector defined in the reference frame of the stationary room or arena, to assess behavioral flexibility in male Long-Evans rats (Svoboda et al. 2015). This investigation showed that MK-801 disrupted the set-shifting task at the dose of 0.1 mg/kg, although it failed to impair reversal learning. Another study of this research group demonstrated that acute MK-801 administration caused cognitive flexibility impairments in attentional set-shifting task in rats. Moreover, optogenetic stimulation of parvalbumin-positive interneurons in the prefrontal cortex and hippocampus rescued the performance (Patrono et al. 2023). Alternative often-used task to assess cognitive flexibility in rodents is the probabilistic reversal learning task (Bartolo and Averbeck 2020; Savolainen et al. 2021). In the study of Latuske et al. (2022), this paradigm was modified to include two distinct parts: performance–based probabilistic reversal learning (representing the standard version of the task) and time–based probabilistic reversal learning. Interestingly, the injection of MK-801 (0.045 mg/kg, *sc*) in Lister-hooded rats, 15 min before the behavioral testing, caused only mild, transient deficits in the first part of the task, while this effect was stronger in the second part, in which MK-801 induced sustained reversal learning deficits (Latuske et al. 2022). These results were consistent with another study, in which performance–based probabilistic reversal learning was only partially affected by the low doses of MK-801 (0.025, 0.05 mg/kg), whereas the higher dose (0.1 mg/kg) completely inhibited reversal learning (van der Meulen et al. 2003).

As previously mentioned, the touchscreen paradigm is another test to assess cognitive flexibility in rodents and is currently gaining increasing popularity. According to the study by Helene Richter et al. (2014), touchscreens tests performed by mice have a close analogy to the human version of this test. Hence, they were directly translated from human procedures, in contrast to other set-shifting paradigms. The administration of MK-801 in Lister-hooded rats impaired performance in different tasks measured in the touchscreen test, such as the location discrimination task and paired associates learning task (Kumar et al. 2015). In the first task, rats treated with MK-801 (0.05 and 0.75 mg/kg, *ip*) showed a decrease in the total number of reversals. MK-801 (0.025, 0.05, 0.075 mg/kg, *ip*) also robustly disrupted performance in the paired associates learning task. Additionally, in this study, MK-801 impaired working memory (Kumar et al. 2015). The higher dose of MK-801 (0.1 mg/kg, *sc*) caused cognitive flexibility impairments in the touchscreen location discrimination reversal task in male C57BL/6 J mice (Graf et al. 2018).

### MK-801 and attentional processing

Attention, as another executive function, refers to the animal’s ability to detect and respond to a salient stimulus by selectively focusing on it amidst numerous other stimuli present in the environment, demanding quick neuronal response (Katsuki and Constantinidis 2014). Due to the that fact, that stimuli processing is a very complex phenomena, it is hard to separate attention from another executive functions (R. C. K. Chan et al. 2008; Norman and Shallice 1986). As mentioned in the review of Harris and Thiele (2011), the term attention covers both ‘top-down attention’, which is induced by the anticipation or expectation of important stimulus, and ‘bottom-up attention’, where attention is draw by the physical properties of a important cue. The ability to ignore the irrelevant stimuli can be defined as ‘latent inhibition’ (MCLAURIN et al. 1963; Weiner and Arad 2009), which is being referred to normal, adaptive modulation of associative learning (Feifel and Shilling 2013). This phenomenon occurs when pre-exposure to a neutral and inconsequential conditioned stimulus reduces the response to subsequent conditioned stimuli when paired with reinforcement, compared to a novel reinforcement (Weiner 2010). NMDA receptor blockade by MK-801 at the doses of 0.1 and 0.2 mg/kg administered *ip* reduced latent inhibition in male Wistar rats in the thirst-motivated conditioned emotional response procedure. In contrast, relatively low dose of dizocilpine, i.e., 0.05 mg/kg induced different effect – abnormally persistent latent inhibition (Gaisler-Salomon and Weiner 2003). Moreover, dizocilpine (0.2 mg/kg, *ip*) did not affect latent inhibition when administered after conditioning in male Wistar rats (Traverso et al. 2003). However, it did abolish latent inhibition when it was injected after the pre-exposure phase, which indicated the importance of NMDA receptors in conditioned stimulus processing and memory consolidation in a conditioned taste-aversion paradigm (Traverso et al. 2003).

An alternative test to asses attentional processes in rodents is five-choice serial reaction time task, that is used to examine visuo-spatial attention and impulse control (Robbins 2002). Smith et al. (2011) suggested that within the group of NMDA receptor antagonists, MK-801 demonstrates minimal advantage over ketamine as a model of attention impairment. In this study, male Lister Hooded rats were administered *sc* with MK-801 or other NMDA receptor antagonist and underwent five-choice serial reaction time task procedure. Dizocilpine at the dose of 0.1 mg/kg decreased the accuracy and increased the amount of omissions, whereas correct response latencies increased in the group that received the dose 0.05 mg/kg (Smith et al. 2011). Similarly, in the study of Guidi et al. male Fisher 344 rats treated with MK-801 at the dose of 0.1 mg/kg showed deficits in attention measured in the five-choice serial reversal time task, while administration of lower doses (0.025, 0.05 mg/kg) enhanced the performance in this test (Guidi et al. 2015).

To sum up, the blockade of NMDA receptors induced by the administration of MK-801 produces deficits in most of the mentioned executive functions. Notably, MK-801 effectively impairs working memory in touch-screen tasks at lower doses, which reduces the likelihood of hyperlocomotion. Similarly, using this test, dizocilpine was found to impair cognitive flexibility in both male and female animals. Interestingly, the MK-801 appears to have contradictory effects on latent inhibition in different studies. It ranges from completely abolishing latent inhibition to producing no effects or even leading to abnormally persistent latent inhibition. Similarly, in five-choice serial reaction time task, MK-801 was showed to either exert no effects on this task, impair the performance or even enhance it. A substantial portion of the described studies that investigate the effects of MK-801 on executive functions have neglected to consider the presence of hyperlocomotion and stereotypy, which may be crucial when analyzing the results (Tables 4, 5 and 6).

**Table 4:** Effects of MK-801 on working memory in various behavioral tests in animals

Dose (mg/kg)	Administration route	Acute Adm	Chronic Adm	Species	Sex	Developmental stage on the day of experiment	Memory deficits	Reference
1. Morris water maze								
0.25	*sc*	-	P5-14	Sprague–Dawley rats	M	adolescent	±	(Su et al. 2014)
						adult	+	
0.25	*sc*	-	P5-14	Sprague–Dawley rats	F	adolecent	±	(Su et al. 2011)
						adult	+	
2. Odor span task								
0.03	*ip*	+	-	Sprague–Dawley rats	M	adult	-	(MacQueen et al. 2011), (Galizio et al. 2013), (Galizio et al. 2019)
0.1							+	
0.17							+	
0.3							-	
3. Spontaneous alternation								
0.1	*sc*	-	P7-10	Sprague–Dawley rats	M	adult	+	(Mark Renato Stefani & Moghaddam 2005)
4. Radial arm maze								
1	*ip*	-	P6-10	Wistar rats	F	adolescent	+	(Nozari et al. 2015)
5. Allothetic place avoidance								
0.12	*sc*	+	-	Sprague–Dawley rats	M	adult	+	(Zemanova et al. 2013)
0.15							+	
6. Touch-screen task								
0.06	*sc*	+	-	Wistar rats	M	adult	+	(Lee et al. 2020)

adm. – administration, *sc* – subcutaneously, P5-14 – postnatal day 5–14, *ip* – intraperitoneally, P7-10 – postnatal day 7–10, P6-10 – postnatal day 6–10, M-male, F-female, -no, + yes,

**Table 5:** Effects of MK-801 on behavioral flexibility in various behavioral tests in animals

Dose (mg/kg)	Administrationroute	Acute Adm	Chronic Adm	Species	Sex	Developmental stage on the day of experiment	Memory deficits	Reference
1. Set-shifting task								
0.1	*sc*	-	GD7-19	Long-Evans rats	M	juvenile	-	(Gallant et al. 2017)
						adult	+	
0.1	*sc*	-	P7-11	Sprague–Dawley rats	M	adult	+	(Mark Renato Stefani and Moghaddam 2005)
0.1	*ip*	+	-	Sprague–Dawley rats	M	adult	+	(Mark R Stefani and Moghaddam 2010)
0.05	n/a	+	-	Long-Evans rats	M	adult	-	(Svoboda et al. 2015)
0.1							+	
2. Touch-screen task								
0.025	*ip*	+	-	Lister-hooded rats	M	adult	+	(Kumar et al. 2015)
0.05							+	
0.075							+	
0.1	*sc*	+	-	C57BL/6J mice	M	adult	+	(Graf et al. 2018)
0.1	*ip*	+	-	C57BL/6J mice	F		+	(Thonnard et al. 2019)
3. Performance-based probabilistic learning								
0.045	*sc*	+	-	Lister-hooded rats	M	adult	±	(Latuske et al. 2022)
4. Time-based probabilistic learning								
0.045	*sc*	+	-	Lister-hooded rats	M	adult	+	(Latuske et al. 2022)
5. Serial reversal task								
0.025	*sc*	+	-	Wistar rats	M	adult	-	(van der Meulen et al. 2003)
0.05							-	
0.1							+	

adm. – administration, *sc* – subcutaneously, P5-14 – postnatal day 5-14, *ip* – intraperitoneally,  P7-10 – postnatal day 7-10, P6-10 – postnatal day 6-10, M-male, F-female, - no, + yes

**Table 6:** Effects of MK-801 on latent inhibition and five-choice serial reversal task

Dose (mg/kg)	Administration route	Acute Adm	Chronic Adm	Species	Sex	Developmental stage on the day of experiment	Attention deficits	Reference
1. Latent inhibition								
0.05	*ip*	+	-	Wistar rats	M	adult	*	(Gaisler-Salomon and Weiner 2003)
0.1							+	
0.2							+	
0.05	*ip*	+	-	Wistar rats	M	adult	*	(Gaisler-Salomon et al. 2008)
0.2	*ip*	+	-	Wistar rats	M	adult	-	(Traverso et al. 2003)
6.25 µg/0.5 μl	*IH*	+	-	Wistar rats	M	adult	-	(Zhang et al. 2000)
2 µg/3 µl	*icv*	+	-	Wistar rats	F	adult	-	(Huang et al., 2017)
					M		-	
2. Five-choice serial reversal task								
0.05	*sc*	+	-	Lister Hooded rats	M	adult	-	(Smith et al. 2011)
0.1							-	
0.01	*ip*	+	-	Lister Hooded rats	M	adult	-	(Benn and Robinson, 2014)
0.03							-	
0.1							-	
3 µg/µl	*intra—IF, PRL*	+	-	Lister Hooded rats	M	adult	-	(Benn & Robinson, 2014)
10 µg/µl		+	-				-	
0.025	*ip*	+	-	Fisher 344 rats	M	young	**	(Guidi et al. 2015)
0.05							**	
0.1							-	
0.025	*ip*	+	-	Fisher 344 rats	M	middle-aged	**	
0.05							**	
0.1							+	
0.25	*ip*	+	-	Sprague–Dawley rats	M	adult	+	(Paine et al., 2007)

adm. – administration, *IF *- infralimbic, *PRL *- prelimbic *ip* – intraperitoneally, *sc* – subcutaneously, *IH –* intrahippocampally, *icv* - intracerebroventricularly, M-male, F-female, - no, + yes, *abnormally persistent, **enhanced

## MK-801's developmental stage-dependent effects on animal cognitive functions

Studies using animal models of cognitive impairments induced by MK-801 frequently focus on investigating compounds with procognitive potential in the context of neurodevelopmental disorders. The ability of MK-801 to influence early developmental stages, enhances its significance and utility in this specific context.

### Recognition memory

Prenatal administration of MK-801 (0.1 mg/kg, *sc*) during gestation day 7–19 in Long-Evans rats caused a notable deficit in novel object preference in adult male specimens, but not juvenile ones, following a 90-min delay. This finding suggests that MK-801 probably impairs animal’s recognition memory (Gallant et al. 2017). A study on neonatal exposure to a higher dose of MK-801 (0.25 mg/kg, twice daily) showed that both adolescent and adult Sprague–Dawley rats, treated postnatally between 5 and 14 days after birth, exhibited deficits in recognition memory evaluated in object and object-in-context recognition tasks (J. T. Li et al. 2015). However, in a murine model, neonatal MK-801 exposure (0.1 mg/kg, *ip*) in distinct temporal windows of administration (postnatal days 7–14 and 11–15) demonstrated different behavioral effects in adult animals (Plataki et al. 2021). Plataki et al. discovered that both male and female C57BL/6 mice, from the group treated on postnatal days 7–14, showed impaired memory in the object, object-to-place, and temporal recognition tasks. In contrast, mice treated on postnatal days 11–15 failed only the temporal recognition test and no differences were observed between male and female animals (Plataki et al. 2021). The study conducted on male Sprague – Dawley rats investigated the effects of chronic MK-801 administration at various doses (0.1, 0.3, 0.5 mg/kg) in postnatal days 7–21. Subsequently, rats underwent four weeks of social isolation during childhood and then five days of social housing. Animals chronically treated with 0.5 mg/kg of MK-801 in infancy exhibited long-term visual memory impairments during adolescence, as assessed in the object recognition test (Liu et al. 2017a, b). Additionally, social isolation did not significantly influence recognition memory (Liu et al. 2017a, b). Conversely, another study using the same model indicated that recognition memory impairment occurred only when the treatment with MK-801 (0.2 mg/kg) was combined with social isolation. In this investigation, dizocilpine was administered in male Sprague–Dawley rats during postnatal days 7–10 and animals were examined for the drug effects using the object recognition test during adulthood (Lim et al. 2012). Although it is interesting to note that repeated low-doses of MK-801 (0.05, 0.1, 0.2 mg/kg, once daily for 14 days, *ip*) block NMDA receptors in adult, male Sprague Dawley rats, causing significant recognition memory deficits in object-in-context recognition task 24 h after the last drug administration but not in the object recognition task (Ji Tao Li et al. 2011). Following 14-day period from administration, recognition memory impairments were reported only in the group treated with a dose of 0.2 mg/kg, as assessed in the object recognition task (Ji Tao Li et al. 2011). Recently, MK-801 was also used in modeling attention deficit hyperactivity disorder in male Swiss albino mice, where treatment with dizocilpine at the dose of 1 mg/kg on postnatal days 7, 14, or 21 induced recognition memory deficits in adolescence and adulthood, assessed in the object recognition test (Kawade et al. 2020). Moreover, adolescent specimens treated on postnatal days 7 and 14, as well as the adult group treated on postnatal day 7, developed hyperactivity. Interestingly, this study also reported a loss of mature neurons in the prefrontal cortex in the adolescent group treated on postnatal day 7 (Kawade et al. 2020).

### Spatial memory

Chronic treatment with MK-801 in male Wistar rat pups during second and third postnatal weeks resulted in impaired spatial learning in the radial maze test in adulthood (Furuie et al. 2019). Furthermore, significant impairments in spatial reference memory were observed in the group treated during both second and third weeks. In contrast the group treated only during the second week showed only mild impairment. Surprisingly, the group treated exclusively during the third week did not demonstrate impairments either in spatial working or spatial reference memory in adulthood (Furuie et al. 2019). Earlier studies on the effects of neonatal chronic administration of MK-801 in rodents, also showed its negative impact on spatial learning and memory (Gorter and de Bruin 1992; Kawabe et al. 2007), as well as affected protein translation, synapse formation, hippocampus–dependent learning, and neuronal development (Elhardt et al. 2010). Conversely, *ip* administration of a low dose of MK-801 (0.25 mg/kg) for 28 days did not affect the spatial working memory of adult male Wistar rats in the Morris water maze (J.-T. Li et al. 2013). Similarly, the study of Uttl et al. (2018) showed that 2-week administration of MK-801 (0.5 mg/kg) in adolescence and early adulthood had no significant effects on working spatial memory assessed in the Morris water maze in male Wistar rats. However, it did impair the spatial working memory in male Long-Evans rats treated from the postnatal day 60, but memory impairment in Long-Evans rats was not associated with the changes in NMDA receptor subunits levels (Uttl et al. 2018).

### Working memory

Numerous studies are focused on the effects of MK-801 on different neurodevelopmental stages in the aspect of working memory. Male Sprague–Dawley rats treated with MK-801 (0.1 mg/kg, *sc*) during postnatal days 7 to 10, a critical period for the development of circuits involving the frontal cortex, caused lasting working memory and cognitive impairments in adult rats assessed in spontaneous alteration and cognitive set-shifting procedures, respectively (Mark Renato Stefani and Moghaddam 2005). Another study showed that neonatal administration of MK-801 (0.25 mg/kg, *sc*) induced spatial working memory deficits in Sprague–Dawley rats assessed in the Morris water maze (Su et al. 2014). Remarkably, this effect was prominent during adulthood, more moderate during adolescence and was ameliorated by the administration of acetylcholinesterase inhibitor galantamine in the adult stage (Su et al. 2014). Su et al. (2011) obtained similar effects in an equivalent study on female specimens, showing that MK-801 administration caused mild working memory impairments during adolescence and pronounced in adulthood (Su et al. 2011). Another study on female Wistar rats indicated that repeated administration of MK-801 (1 mg/kg, *ip*) during postnatal days 6 to 10 resulted in working memory deficits assessed in the radial arm maze in adolescence, and this effect was not restored in the group of animals housed in the enriched environment (Nozari et al. 2015).

### Behavioral flexibility

An inconsiderable amount of research shows the impact of chronic MK-801 treatment on cognitive flexibility. The study described above in the section dedicated to recognition memory investigated the influence of chronic, prenatal (gestation day 7–19) administration of dizocilpine (0.1 mg/kg, *sc*) on cognitive flexibility in adult specimens (Gallant et al. 2017). In this study, cognitive flexibility was measured in the plus-maze-based set-shifting procedure in male Long-Evans rats. Adult rats, prenatally treated with MK-801, revealed the impaired acquisition of the new rule, and it appeared to be driven by the regression of previously learned behavior (Gallant et al. 2017). Another investigation on the effects of transient MK-801 administration (0.1 mg/kg, *sc*) in postnatal days 7–10 in male Sprague–Dawley rats showed impaired cognitive flexibility assessed in the cognitive set-shifting procedure (Mark Renato Stefani and Moghaddam 2005). Interestingly, animals also demonstrated deficits in working memory, but MK-801 failed to impair recognition memory (Mark Renato Stefani and Moghaddam 2005). The effects of repeated administration of dizocilpine (0.1 mg/kg, *ip*) on performance in a novel, cognitively demanding, pairwise discrimination and reversal learning task was examined in the study of Thonnard et al. (2019) conducted on C57BL/6 J female mice. The results indicated that MK-801 did not affect initial acquisition learning but impaired reversal learning. Interestingly, the abovementioned results contradict studies using different paradigms (Latuske et al. 2022; Patrono et al. 2023).

MK-801 serves as a useful compound to study disruptions in NMDA-receptor-mediated cognitive functions in the context of neurodevelopmental changes. Gestational administration of MK-801 results in impaired recognition memory and behavioral flexibility during adulthood, while these effects are absent in juvenile animals. Moreover, when MK-801 was administered to animals during postnatal days 7–10, it caused a decline in behavioral flexibility in adult animals. This specific period appears to be crucial for development, as evidenced by similar outcomes observed when assessing its effects on spatial and working memory. The presence or absence of deficits caused by MK-801 in all reviewed cognitive functions was dependent on the specific time window during the prenatal period when dizocilpine was administered. Tables 1, 2, 4 and 5 include the studies showing the developmental impact of MK-801.

## Discussion and conclusions

**Fig. 1:**
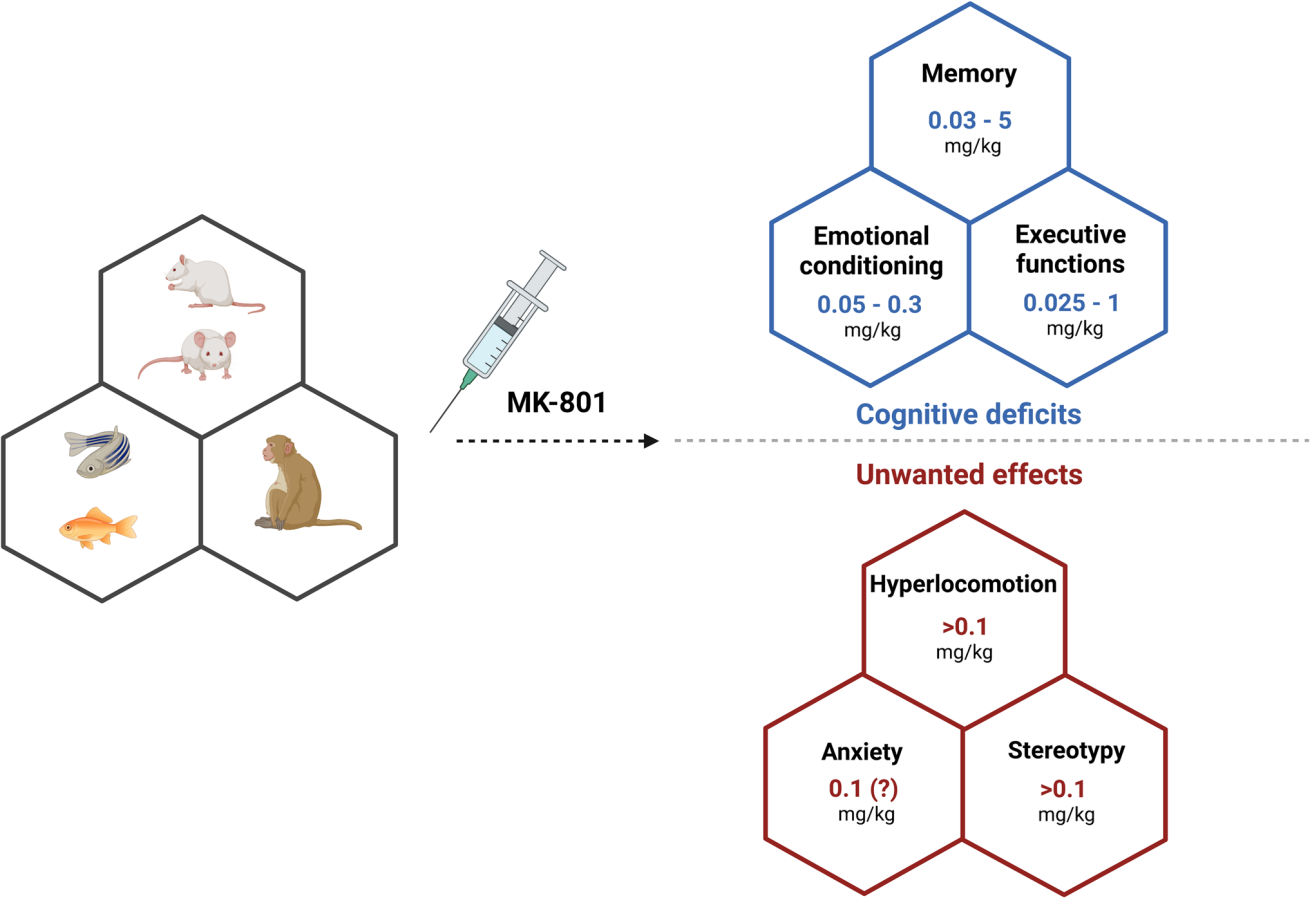
An overview of the doses of MK-801 employed in cognitive studies, along with their potential to induce undesirable effects within these research contexts. Created with BioRender.com

MK-801, a non-competitive NMDA receptor antagonist, is broadly used in modeling cognitive impairments while testing various procognitive therapeutics. It is considered as one of the most potent NMDA antagonist (Brandão-Teles et al. 2017), and studies demonstrated its high equipotency across all NMDA receptor subtypes, distinguishing it from phencyclidine or ketamine (Dravid et al. 2007; Lodge and Mercier 2015). Dizocilpine was shown to deteriorate cognitive functions, including recognition memory, spatial memory, emotional memory, as well as executive functions like working memory or cognitive flexibility (Fig. 1). However, the exact effects of this compound on memory highly depend on numerous factors, including developmental stage, sex, animal species or strain, timing of administration in relation to memory formation, dosage, and even the specific behavioral test employed.

Despite the number of pharmacological advantages of MK-801, some properties of this agent are controversial when comparing the results of diverse studies. MK-801-induced hyperactivity is an apparent and well-known problem in behavioral studies while using this compound to induce cognitive impairments, although different studies report different doses when such disruptions in locomotor activity arise (Fig. 1). This problem was addressed in the study conducted by Liu et al. (2017a, b), where the authors established that a dose of 0.03 mg/kg effectively induces ‘pure’ cognitive impairment while dissociating it from accompanying side effects. Nevertheless, the majority of research indicates that the onset of hyperlocomotion, or stereotypy, generally commences at dosage levels exceeding 0.1 mg/kg. Also, it has been noted that while authors of various studies investigating MK-801-induced memory deficits do consider the aspect of sensorimotor activity, the methods used to address it may not be sufficient. The authors propose that a valid and effective method to evaluate sensorimotor gating is a prepulse inhibition model (Liu et al. 2017a, b). Considering these results, the studies reviewed in their paper show a remarkably wide range of doses being used in various studies. Therefore, it is important to standardize the doses of MK-801, used in the aspect of various types of memory or executive function, to enable straightforward cross-study comparisons, as well as to establish doses ‘free’ of hyperactivity induction. Moreover, it is worth noting that detecting such disruptions in locomotor activity may affect the reliability of the results, and some studies do not cover this aspect at all. In Fig. 1, we have encapsulated the range of doses that have been observed to interfere with cognitive processes, along with those that have manifested undesirable side effects within the ambit of cognitive studies. Our analysis indicates that doses of MK-801 up to 0.1 mg/kg should not induce the aforementioned undesirable effects. Consequently, we suggest that researchers might want to concentrate their efforts on these dosage levels when conducting cognitive studies.

A large body of evidence indicates that NMDA receptors in the ventral hippocampus are involved in processes underlying anxiety-like behaviors (Bannerman et al. 2014; Barkus et al. 2010; Deacon 2011; Niewoehner et al. 2007). Considering that the hippocampus is involved in memory processes, it is also worth mentioning that the pharmacological blockade of NMDA receptors with MK-801 may reduce anxiety in rodents (Amani et al. 2019; Dunn et al. 1989; Engin et al. 2009; Xie and Commissaris 1992). However, some studies reported opposite results (Abuhamdah et al. 2016; Ennaceur et al. 2011). Therefore, it is worth addressing the problem of the anxiolytic or anxiogenic potential of MK-801, as it may influence the results.

Another challenging aspect of using MK-801 in modeling cognitive impairments is the basis of a selected cognitive function itself. The role of the NMDA receptor in certain cognitive functions is relatively ambiguous, and non-competitive antagonist MK-801 may successfully impair these functions to some extent. It is worth mentioning that MK-801 influences not only glutamatergic transmission. Several studies suggest that dizocilpine also affects GABAergic (Auger and Floresco 2017; Roenker et al. 2012; Thomases et al. 2013) and dopaminergic transmission (Bartsch et al. 2015; Saoud et al. 2021; Tsukada et al. 2005). Even when investigating the same type of cognitive function, there may be differences between the results of behavioral tests that use MK-801 as a cognitive impairer. Therefore, it is important to standardize the behavioral procedures used in pre-clinical studies to facilitate better cross-study comparisons.

To sum up, MK-801 was reported to induce impairments of encoding and consolidation of different types of memory, not only in murine models but also in other species, administered both chronically and systematically. Furthermore, MK-801 can induce long-term effects in early developmental stages in both male and female specimens, which may be valuable for preclinical studies. The potential presence of hyperlocomotion, stereotypy or anxiogenic/anxiolytic effects remains a challenge, as they can mask the effects of cognitive impairments, making it difficult to distinguish and may lead to false-positive results. Despite the above controversies, dizocilpine, as one of the most potent NMDA receptor antagonists, offers a cost-effective alternative compared to, for instance, the use of genetically ablated animals, thereby enabling its broad application. Moreover, MK-801 is also useful in examining molecular characteristics associated with positive, negative, and cognitive symptoms of schizophrenia, rendering it a highly universal compound with multifaceted applications. It is critical for future studies to incorporate the concerns raised in this review to improve the comparability and overall validity across studies.
